# Pulmonary neuroendocrine cells at the airway neuroimmune interface: a bibliometric analysis

**DOI:** 10.3389/fmed.2026.1953372

**Published:** 2026-10-14

**Authors:** Jianfeng Jiang, Zhiyuan Zhao, Chen Huang, Yingxiang Cheng, Haizhen Ding

**Affiliations:** 1 Department of Pediatrics, The Fourth Affiliated Hospital of Jiangsu University, Zhenjiang, People's Republic of China; 2 Department of Anesthesiology, The Fourth Affiliated Hospital of Jiangsu University, Zhenjiang, People's Republic of China

**Keywords:** airway chemosensation, allergic asthma, bibliometric analysis, epithelial plasticity, neuroepithelial bodies, neuroimmune regulation, neuropeptides, pulmonary neuroendocrine cells

## Abstract

**Background:**

Pulmonary neuroendocrine cells (PNECs) and neuroepithelial bodies (NEBs) are specialized airway epithelial structures involved in environmental sensing, neuroendocrine secretion, epithelial regulation, and inflammatory responses. However, the bibliometric structure, thematic evolution, and emerging neuroimmune orientation of PNEC/NEB research at the airway neuroimmune interface have not been quantitatively characterized.

**Methods:**

Publications from 1990 to 2025 were retrieved from the Web of Science Core Collection (WoSCC) and Scopus using a predefined search strategy. After database merging, deduplication, and bibliographic standardization, 595 English-language articles and reviews were included. Scopus was predefined as the primary source of document-level citation counts, with WoSCC counts used only for publications not indexed in Scopus; database-specific and maximum-count approaches were evaluated in sensitivity analyses. Bibliometrix, VOSviewer, and CiteSpace were used to analyze publication trends, geographic and institutional contributions, author collaboration, journal and reference co-citation, keyword clustering, temporal evolution, and citation bursts. Representative experimental studies were used to contextualize the major bibliometric themes.

**Results:**

The included publications accumulated 25,399 citations under the predefined primary citation-count rule, with research activity increasing after 2019 and reaching a maximum of 28 publications in 2024. Citation-based rankings were robust across database-specific and alternative counting approaches, and the top 10 publications were unchanged under the maximum-count sensitivity analysis. The United States contributed the largest number of publications, while the University of Toronto and University of Antwerp were among the leading institutions. Representative experimental studies were used to contextualize these bibliometric patterns by describing mediator-specific interactions among PNECs and epithelial, neural, and immune compartments. The thematic structure comprised pulmonary neuroendocrine signaling, airway chemoreception, neuropeptide processing, and lung development, among others. Temporal and burst analyses indicated that early research focused on NEB morphology, bombesin/GRP, oxygen sensing, and airway chemoreception, whereas later studies increasingly addressed PNEC-specific biology, epithelial plasticity, hyperplasia, allergic inflammation, and neuroimmune signaling.

**Conclusion:**

PNEC research has expanded from morphological and chemosensory characterization toward an integrated framework involving epithelial plasticity, airway functional responses, and neuroimmune communication. Human-relevant models, spatially resolved analyses, and cell- and mediator-specific experiments will be essential for defining the context-dependent functions and therapeutic relevance of PNECs.

## 1 Introduction

Pulmonary neuroendocrine cells (PNECs) represent a rare but highly specialized epithelial lineage distributed throughout the airway epithelium, occurring either as solitary cells or organized clusters known as neuroepithelial bodies (NEBs). These cells exhibit characteristic neuroendocrine features, including dense-core secretory granules and expression of markers such as chromogranin A (CHGA), synaptophysin (SYP), and achaete-scute family bHLH transcription factor 1 (ASCL1) (1). Although PNECs constitute only a minor fraction of airway epithelial cells, recent single-cell transcriptomic studies have revealed substantial molecular heterogeneity and functional diversity within this population (2). Traditionally regarded as airway neuroendocrine structures involved in sensory regulation, PNECs have been shown to detect environmental changes, including oxygen availability and chemical stimuli, and translate these signals into local cellular responses (3, 4). Through the regulated release of neuropeptides and neurotransmitters, PNECs establish communication networks with surrounding epithelial and neural compartments, suggesting broader roles in maintaining airway homeostasis (5). These discoveries have shifted the perception of PNECs from passive neuroendocrine markers toward active regulators of airway physiology.

Beyond their classical sensory functions, PNECs are increasingly recognized as important regulators of lung development, epithelial plasticity, and tissue repair. During pulmonary development, PNEC differentiation is governed by conserved neuroendocrine lineage programs in which ASCL1 has an essential role (6). Experimental studies have identified stem-like properties in PNECs and demonstrated that their behavior is regulated by epithelial niche signals, supporting a potential role in airway epithelial maintenance and regeneration (7). Furthermore, accumulating evidence suggests that PNECs serve as potential mediators linking environmental sensing with immune responses. By releasing signaling molecules such as calcitonin gene-related peptide (CGRP), serotonin, *γ*-aminobutyric acid (GABA), and other neuroactive mediators, PNECs may regulate interactions among epithelial cells, sensory nerves, and immune populations (2, 5). Recent studies have particularly implicated PNECs in allergic airway inflammation, where neuroendocrine signaling contributes to type 2 immune responses and airway remodeling (8). Experimental evidence further indicates that PNECs can translate airway signals into local immune responses through neuropeptide-dependent communication (9). These findings have introduced a new conceptual framework in which PNECs function as an airway neuroimmune interface.

Despite rapid advances in PNEC biology, current knowledge remains fragmented across multiple research fields, including developmental biology, respiratory physiology, epithelial regeneration, neurobiology, inflammation, and pulmonary neuroendocrine disorders. Existing studies have provided important insights into individual aspects of PNEC biology, including oxygen sensing, epithelial repair, and inflammatory regulation. However, the bibliometric evolution of PNEC/NEB research at the airway neuroimmune interface and the emergence of neuroimmune concepts within this literature remain poorly defined. PNEC-related research encompasses diverse biological contexts, ranging from normal airway homeostasis to pathological conditions such as diffuse idiopathic pulmonary neuroendocrine cell hyperplasia (DIPNECH) and pulmonary neuroendocrine tumors (10). This diversity makes it challenging to distinguish physiological functions from disease-associated alterations. Although recent studies have highlighted the potential involvement of PNECs in immune regulation and inflammatory diseases, a quantitative overview integrating research trends, knowledge structures, and relevant mechanistic developments is still lacking. Therefore, a quantitative bibliometric assessment of this literature landscape is needed to clarify its research evolution and identify future scientific priorities.

Bibliometric analysis provides a quantitative approach to evaluate research productivity, intellectual structure, collaboration networks, and emerging scientific trends through publication patterns, keyword evolution, and citation relationships. Bibliometric findings can be contextualized using representative experimental studies to relate quantitative research patterns to biological developments. Therefore, this study performed a multi-database bibliometric analysis of PNEC research at the airway neuroimmune interface from 1990 to 2025, integrating Web of Science Core Collection (WoSCC) and Scopus datasets with visualization and network-analysis approaches. We aimed to characterize publication trends, major contributors and knowledge structures, trace the temporal evolution of airway sensory, developmental, neuropeptide, and neuroimmune-related themes within this interface-focused literature, and contextualize the major bibliometric patterns using representative foundational and experimental studies of PNEC-associated airway biology.

## 2 Materials and methods

### 2.1 Data sources and search strategy

A predefined bibliographic database search was conducted in the Web of Science Core Collection and Scopus to identify publications concerning PNECs/NEBs in relation to airway neuroimmune, inflammatory, and neuropeptide-associated biology. The publication period was restricted to January 1, 1990-December 31, 2025. Eligible document types were original articles and reviews published in English. Records meeting the predefined database, publication-period, language, and document-type criteria were retained after deduplication and bibliographic verification; no additional title/abstract or full-text eligibility screening was performed.

Database-specific search strategies combined three conceptual components: PNEC- or NEB-related terms, pulmonary or airway terms, and neuroimmune-, inflammatory-, or neuropeptide-related terms. The latter terms were deliberately included to maintain consistency between literature retrieval and the study focus on the neuroimmune roles and signaling functions of PNECs/NEBs in the airway. The resulting dataset was therefore intended to represent the literature most relevant to this thematic focus rather than the entirety of PNEC-related research. The complete search strings are provided in Supplementary Material 1. All searches and bibliographic exports were completed on June 30, 2026 to minimize variation caused by database updates. WoSCC records were exported as plain-text files containing full records and cited references, whereas Scopus records were exported in CSV and RIS formats containing citation information, abstracts, keywords, affiliations, and cited references.

### 2.2 Data merging and preprocessing

Bibliographic data were processed in R version 4.6.0 using the bibliometrix package version 4.3.1. WoSCC and Scopus files were converted into standardized bibliographic data frames using the *convert2df()* function and subsequently merged using *mergeDbSources()* with duplicate removal enabled. The WoSCC search yielded 343 records and the Scopus search yielded 484 records. Of the 827 records retrieved initially, 232 duplicates were removed, leaving 595 publications in the final dataset. Automatic deduplication was followed by manual verification using DOI as the primary identifier. Records without a DOI or with inconsistent DOI information were compared sequentially according to normalized title, first author, publication year, journal, volume, and page or article number. Online-first and final-version records were treated as the same publication when their bibliographic information referred to an identical article.

Author names, institutional affiliations, countries or regions, journal titles, keywords, and cited references were standardized before analysis. Obvious spelling variants, singular and plural forms, abbreviations, and synonymous terms were harmonized using a manually constructed thesaurus. Variants of “pulmonary neuroendocrine cells,” “PNECs,” “pulmonary neuroendocrine cell,” “neuroepithelial body,” and “neuroepithelial bodies” were normalized where appropriate. Institutional variants were consolidated when they could be assigned reliably to the same organization, whereas department-level affiliations were retained as separate units when their parent institution could not be determined unambiguously. Cited-reference variants were matched using DOI, author, publication year, journal, volume, and page information.

### 2.3 Citation indicators and descriptive bibliometric analysis

Scopus was predefined as the primary source of document-level citation counts because it covered a larger proportion of the retrieved literature in the present dataset (484 records in Scopus vs. 343 in WoSCC). Accordingly, the Scopus citation count was used for publications indexed in Scopus, whereas the WoSCC citation count was used only for publications not indexed in Scopus. Citation counts from the two databases were never summed. The resulting values represent document-level citation counts under this predefined operational rule rather than database-independent unified citation counts. Sensitivity analyses were performed to determine whether the principal citation-based findings depended on database choice or counting rule. For a separate cross-database sensitivity analysis, a conservatively matched subset of 217 records with unambiguous one-to-one correspondence between the WoSCC and Scopus source files was used solely to assess citation-count concordance; this subset was independent of the database-merging procedure used to determine the final 595-publication dataset. Rank concordance between WoSCC and Scopus citation counts was assessed using Spearman's rank correlation coefficient, and the direction and magnitude of paired differences were summarized descriptively. The top-10 publications derived separately from the WoSCC and Scopus source datasets were compared using top-10 overlap. The primary rule was also compared with a maximum-count sensitivity approach, in which the higher WoSCC or Scopus count was retained for each citation-comparable cross-database pair. Robustness was evaluated using Spearman's rank correlation, top-10 overlap, the number and proportion of affected publications, and the absolute and relative changes in total citations. Complete results are presented in Supplementary Table S2.

Author H-indices were calculated within the final 595-publication dataset and were therefore treated as local H-indices rather than authors' career-wide database H-indices. The h-index of an author was defined as the largest value h for which at least h publications included in the dataset had each received at least h citations. Publication-level citation rankings, annual citation totals, and the publications with the highest citation counts presented in Table 1 were calculated using the predefined primary citation-count rule.

**Table 1:** Leading co-cited references and publications with the highest citation counts in PNEC/NEB research at the airway neuroimmune interface from 1990 to 2025. The first section presents the leading references according to co-citation frequency and network degree, with degree defined as the number of direct links connected to a reference in the co-citation network. The second section presents the publications with the highest document-level citation counts under the predefined primary citation-count rule. Scopus citation counts were used for publications indexed in Scopus, whereas WoSCC counts were used for publications not indexed in Scopus; citation counts were never summed across databases. Sensitivity analyses of database-specific and alternative citation-count approaches are presented in Supplementary Table S2. Titles were shortened only where necessary for table layout.

Rank	First author (year)	Publication	Journal	Bibliometric indicator
A. Leading co-cited references				
1	Branchfield K (2016)	Pulmonary neuroendocrine cells function as airway sensors to control lung immune response	*Science*	19 co-citations; degree 13
2	Cutz E (2013)	Recent advances and controversies on the role of pulmonary neuroepithelial bodies as airway sensors	*Semin Cell Dev Biol*	12 co-citations; degree 11
3	Song H (2012)	Functional characterization of pulmonary neuroendocrine cells in lung development, injury, and tumorigenesis	*Proc Natl Acad Sci U S A*	9 co-citations; degree 9
4	Brouns I (2009)	Neurochemical pattern of the complex innervation of neuroepithelial bodies in mouse lungs	*Histochem Cell Biol*	8 co-citations; degree 13
5	De Proost I (2009)	Purinergic signaling in the pulmonary neuroepithelial body microenvironment unraveled by live-cell imaging	*FASEB J*	5 co-citations; degree 6
6	Gorshtein A (2012)	Diffuse idiopathic pulmonary neuroendocrine cell hyperplasia and associated lung neuroendocrine tumors	*Cancer*	5 co-citations; degree 8
7	Baral P (2018)	Nociceptor sensory neurons suppress neutrophil and gamma-delta T-cell responses in bacterial lung infection	*Nat Med*	5 co-citations; degree 5
8	Nassar AA (2011)	Diffuse idiopathic pulmonary neuroendocrine cell hyperplasia: a systematic overview	*Am J Respir Crit Care Med*	5 co-citations; degree 3
9	Schnorbusch K (2013)	GABAergic signaling in the pulmonary neuroepithelial body microenvironment	*Histochem Cell Biol*	4 co-citations; degree 1
10	Morrisey EE (2010)	Preparing for the first breath: genetic and cellular mechanisms in lung development	*Dev Cell*	4 co-citations; degree 8
B. Publications with the highest citation counts				
1	Brambilla E (2001)	The new World Health Organization classification of lung tumours	*Eur Respir J*	895 citations under primary rule
2	Warburton D (2000)	The molecular basis of lung morphogenesis	*Mech Dev*	567 citations under primary rule
3	Travis W (2011)	Pathology of lung cancer	*Clin Chest Med*	509 citations under primary rule
4	Giangreco A (2002)	Terminal bronchioles harbor a unique airway stem-cell population at the bronchoalveolar duct junction	*Am J Pathol*	494 citations under primary rule
5	Hewitt R (2021)	Regulation of immune responses by the airway epithelial cell landscape	*Nat Rev Immunol*	460 citations under primary rule
6	Gustafsson B (2008)	Bronchopulmonary neuroendocrine tumors	*Cancer*	432 citations under primary rule
7	Hong K (2001)	CCSP-expressing cells of the NEB microenvironment are critical for epithelial renewal after progenitor depletion	*Am J Respir Cell Mol Biol*	427 citations under primary rule
8	Reynolds S (2000)	Neuroepithelial bodies serve as a reservoir of progenitor cells capable of epithelial regeneration	*Am J Pathol*	395 citations under primary rule
9	Ito T (2000)	Basic helix-loop-helix factors regulate neuroendocrine differentiation of fetal mouse pulmonary epithelium	*Development*	373 citations under primary rule
10	Sui P (2018)	Pulmonary neuroendocrine cells amplify allergic asthma responses	*Science*	369 citations under primary rule

CCSP, club cell secretory protein; NEB, neuroepithelial body; PNEC, pulmonary neuroendocrine cell; WoSCC, Web of Science Core Collection.

Descriptive bibliometric analyses were performed using bibliometrix and Biblioshiny. The analyses included annual publication output, annual citation totals, document-level citation indicators, and the contributions of countries or regions, institutions, authors, and journals. International collaboration was evaluated using single-country publications (SCPs) and multiple-country publications (MCPs). Journal productivity was assessed according to the number of included publications, whereas source co-citation frequency was analyzed separately as an indicator of shared cited-journal usage.

### 2.4 Network, clustering, and temporal analyses

VOSviewer version 1.6.20 was used to construct country, institutional, and author co-authorship networks and cited-author and cited-source co-citation networks. Full counting was applied. In these maps, node size represented publication output or co-citation frequency, links indicated collaboration or co-citation relationships, and total link strength represented the cumulative strength of all links connected to a node. No additional minimum inclusion threshold was imposed because of the relatively limited dataset size, and all eligible items were retained in the corresponding VOSviewer analyses.

CiteSpace version 6.4.1 was used for keyword co-occurrence networks, keyword clustering, reference co-citation analysis, timeline visualization, and burst detection. The period from 1990 to 2025 was divided into 1-year time slices. Keywords and cited references were selected as node types in the corresponding analyses. Nodes were selected using the g-index with a scaling factor of *k* = 25. Pathfinder pruning, pruning of sliced networks, and pruning of the merged network were applied. Initial cluster labels were generated using the log-likelihood ratio method and were refined for clarity according to the representative terms within each cluster, without altering cluster membership. Cluster quality was evaluated using the modularity *Q* value and weighted mean silhouette score. Modularity *Q* reflects the degree of separation between clusters, whereas the silhouette score reflects the internal consistency of items within each cluster; higher values indicate a more clearly defined clustering structure.

Keyword and reference bursts were detected using the Kleinberg burst-detection algorithm. Burst strength represented the magnitude of the temporary increase in keyword occurrence or reference citation frequency, while the beginning and ending years defined the duration of the burst. Annual publication and citation charts and other descriptive figures were prepared using OriginPro version 2024b.

## 3 Results

### 3.1 Literature retrieval and publication dynamics

The literature retrieval, merging, deduplication, and preprocessing procedures are summarized in Figure 1A. Briefly, 343 records from the Web of Science Core Collection and 484 records from Scopus were combined, and 232 duplicates were removed, resulting in a final bibliometric dataset of 595 publications published between 1990 and 2025.

**Figure 1:**
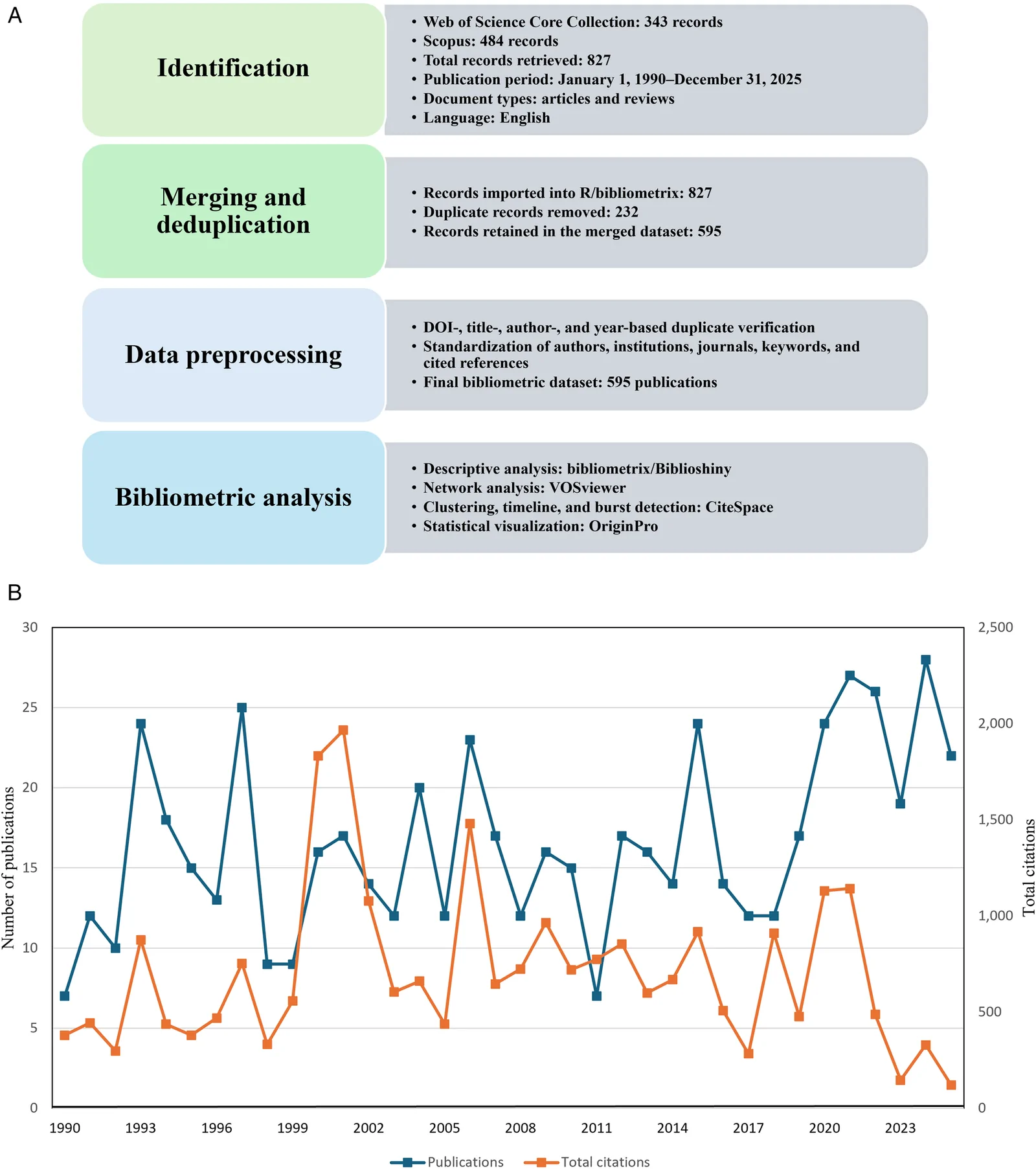
Literature retrieval workflow and publication and citation trends in PNEC/NEB research at the airway neuroimmune interface from 1990 to 2025. **(A)** Workflow of literature retrieval, database merging, deduplication, bibliographic standardization, and bibliometric analysis. Records retrieved from the Web of Science Core Collection and Scopus were merged using R/bibliometrix, resulting in a final dataset of 595 publications. **(B)** Annual publication output and total citations of publications grouped by publication year from 1990 to 2025. Citation values represent cumulative citations received by each publication-year cohort up to the date of data retrieval and were calculated using the predefined primary citation-count rule, with Scopus counts used when available and WoSCC counts used only for publications not indexed in Scopus.

Annual publication output fluctuated across the study period, with a relatively higher level of activity after 2019 (Figure 1B; Supplementary Table 1A). The annual number of publications increased from 17 in 2019 to 24 in 2020, 27 in 2021, and 26 in 2022, before declining to 19 in 2023 and reaching a maximum of 28 in 2024; 22 publications were recorded in 2025. Under the predefined primary citation-count rule, the 595 publications accumulated 25,399 citations, with an average of 42.69 citations per publication. Citation totals grouped by publication year also varied markedly, with the most prominent peaks observed among publications from 2000 to 2001 and 2006.

Citation-count sensitivity analysis showed that the principal citation-based findings were robust to database choice and counting rule (Supplementary Table S2). Among the 217 citation-comparable WoSCC–Scopus pairs, WoSCC and Scopus citation counts showed very strong rank concordance (Spearman's *ρ* = 0.994, *P* < 0.001). Scopus reported a higher citation count for 160 publications (73.7%), the databases reported equal counts for 39 (18.0%), and WoSCC reported a higher count for 18 (8.3%). Median citation counts were 23 in Scopus and 21 in WoSCC, and the median paired difference, calculated as Scopus minus WoSCC, was 2 citations (interquartile range, 0–7). The database-specific WoSCC and Scopus rankings shared 8 of their top 10 publications. The primary and maximum-count approaches produced near-identical rankings (Spearman's *ρ* = 0.9995, *P* < 0.001), and the identity and order of the top 10 publications were unchanged. The maximum-count approach affected only 18 of 595 publications (3.0%) and increased the overall citation total by 35 citations (0.14% of the primary total).

### 3.2 Geographic and institutional contributions

Research output was concentrated in North America, Europe, and East Asia (Figure 2A; Supplementary Table 1B). The United States ranked first with 218 publications, followed by Canada with 57, Belgium with 54, Japan with 43, the United Kingdom with 39, China with 29, Italy with 28, Germany with 24, Spain with 15, and Poland with 15. The United States occupied the largest node in the international collaboration network and maintained extensive links with Canada and several European countries, including the United Kingdom, Belgium, Germany, the Netherlands, Switzerland, France, and Italy. Additional international connections involved Japan, China, Australia, and other participating countries.

**Figure 2:**
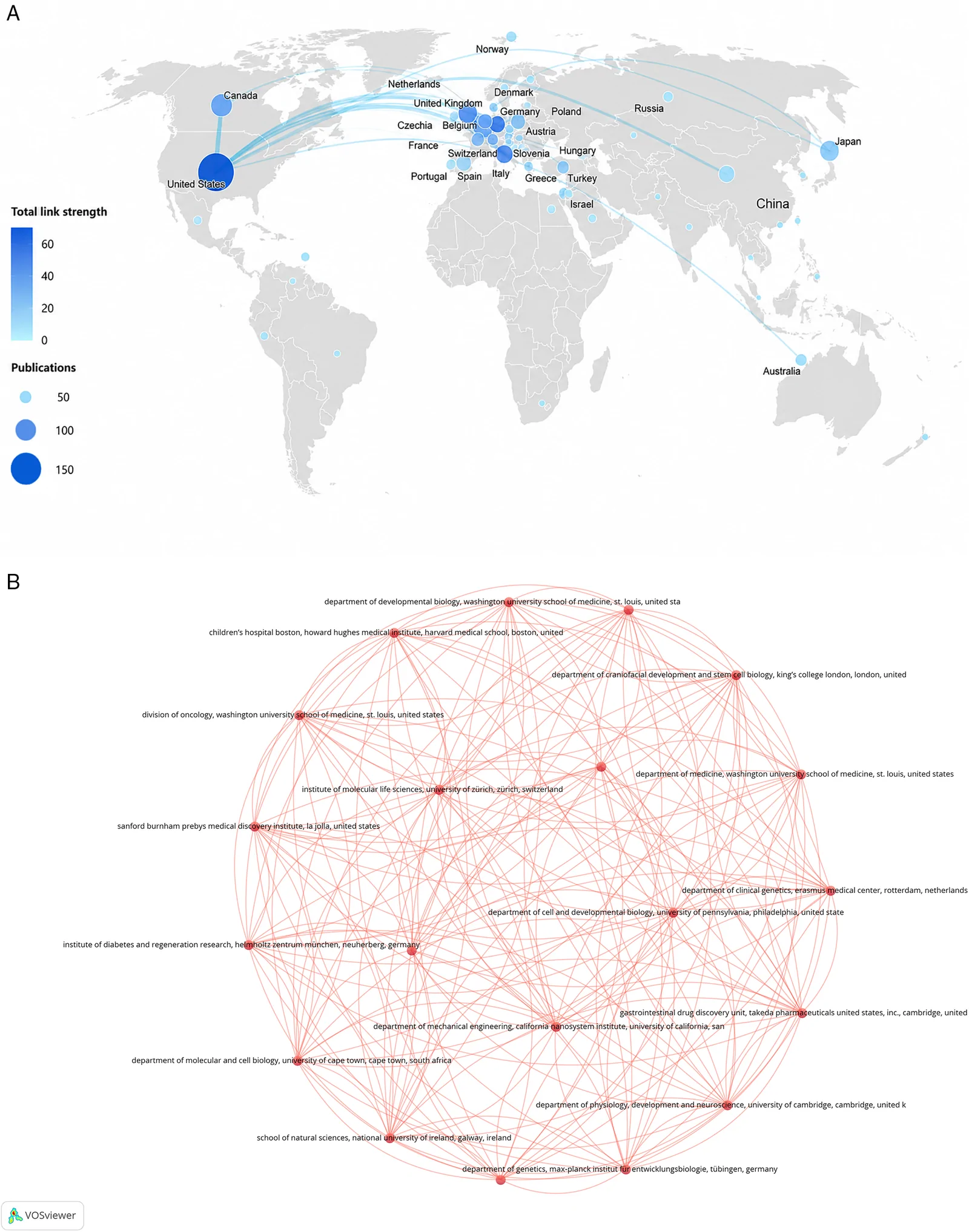
Global distribution and institutional collaboration patterns in PNEC/NEB research at the airway neuroimmune interface from 1990 to 2025. **(A)** Geographic distribution and international collaboration network of publications. Node size represents the number of publications contributed by each country or region, node color indicates total link strength, and connecting lines represent international co-authorship relationships. **(B)** Institutional collaboration network based on author affiliations. Nodes represent institutions or institution-level affiliations, and links indicate collaborative relationships between them. Multiple departments from the same parent institution may appear as separate nodes because affiliation data were retained at the departmental or institutional-unit level.

Most publications from the leading countries were single-country publications. The United States produced 204 single-country and 14 multiple-country publications, while the corresponding values were 52 and 5 for Canada, 54 and 0 for Belgium, 40 and 3 for Japan, 32 and 7 for the United Kingdom, and 27 and 2 for China (Supplementary Table 1B). At the institutional level, the University of Toronto ranked first with 40 publications, followed by the University of Antwerp with 32 and Harvard University with 26 (Figure 2B; Supplementary Table 1C). The remaining institutions in the top 10 were Harvard Medical School (*n* = 20), Harvard University Medical Affiliates (*n* = 20), the University of California (*n* = 20), Baylor College of Medicine (*n* = 17), Duke University (*n* = 14), the Hospital for Sick Children (*n* = 13), and Massachusetts General Hospital (*n* = 12). The collaboration network also contained multiple departmental affiliations from Washington University School of Medicine and other large academic systems.

### 3.3 Author productivity and collaboration patterns

Cutz E was the most productive author, with 47 publications, 1,836 citations, and a local h-index of 27 (Supplementary Table 1D). Adriaensen D ranked second with 41 publications, 1,546 citations, and a local h-index of 22, followed by Timmermans J with 40 publications, 1,425 citations, and a local h-index of 22. The remaining authors in the top 10 were Brouns I (32 publications), Sunday M (27), Yeger H (26), Pan J (21), Pintelon I (20), Lauweryns J (18), and Ito T (17).

The co-authorship network contained several interconnected author groups (Figure 3A). Cutz E occupied a prominent central position and was connected with authors including Sunday M, Yeger H, Pan J, Cuttitta F, and other contributors. Additional clusters were formed around European, North American, and Japanese research groups, with cross-cluster links connecting several of the major author networks.

**Figure 3:**
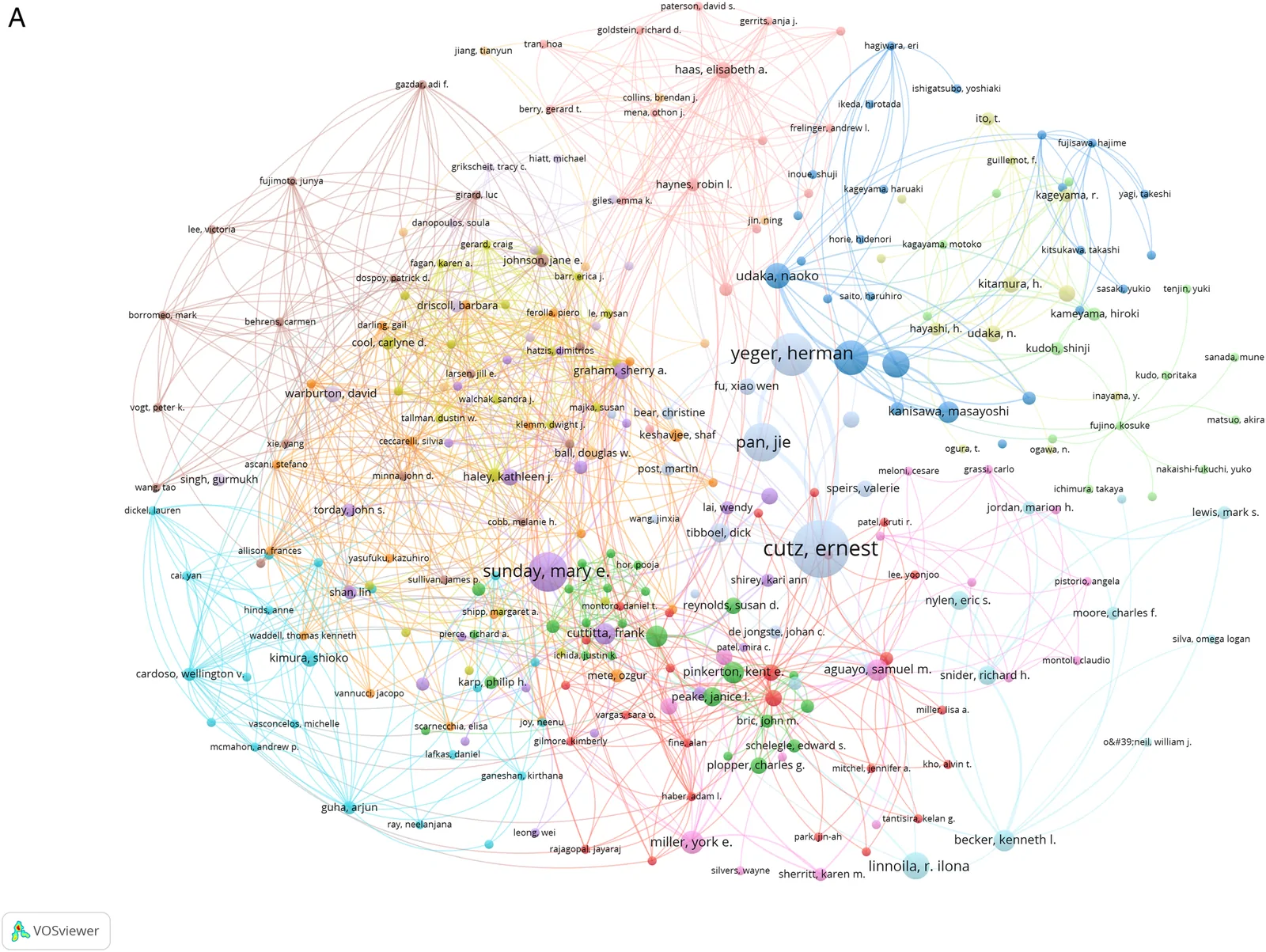
Author productivity, collaboration patterns, and co-citation structure in PNEC/NEB research at the airway neuroimmune interface from 1990 to 2025. **(A)** Author co-authorship network. Nodes represent authors, node size reflects publication output, colors indicate collaboration clusters identified by VOSviewer, and links represent co-authored publications. **(B)** Cited-author co-citation network. Nodes represent cited authors, node size reflects co-citation frequency, colors indicate co-citation clusters, and links indicate that two authors were cited together in the included publications. Co-citation relationships do not represent direct research collaboration. **(C)** Source co-citation network. Nodes represent cited journals, node size reflects source co-citation frequency, colors indicate groups of closely connected sources, and links represent the frequency with which journals were cited together.

**Figure:**
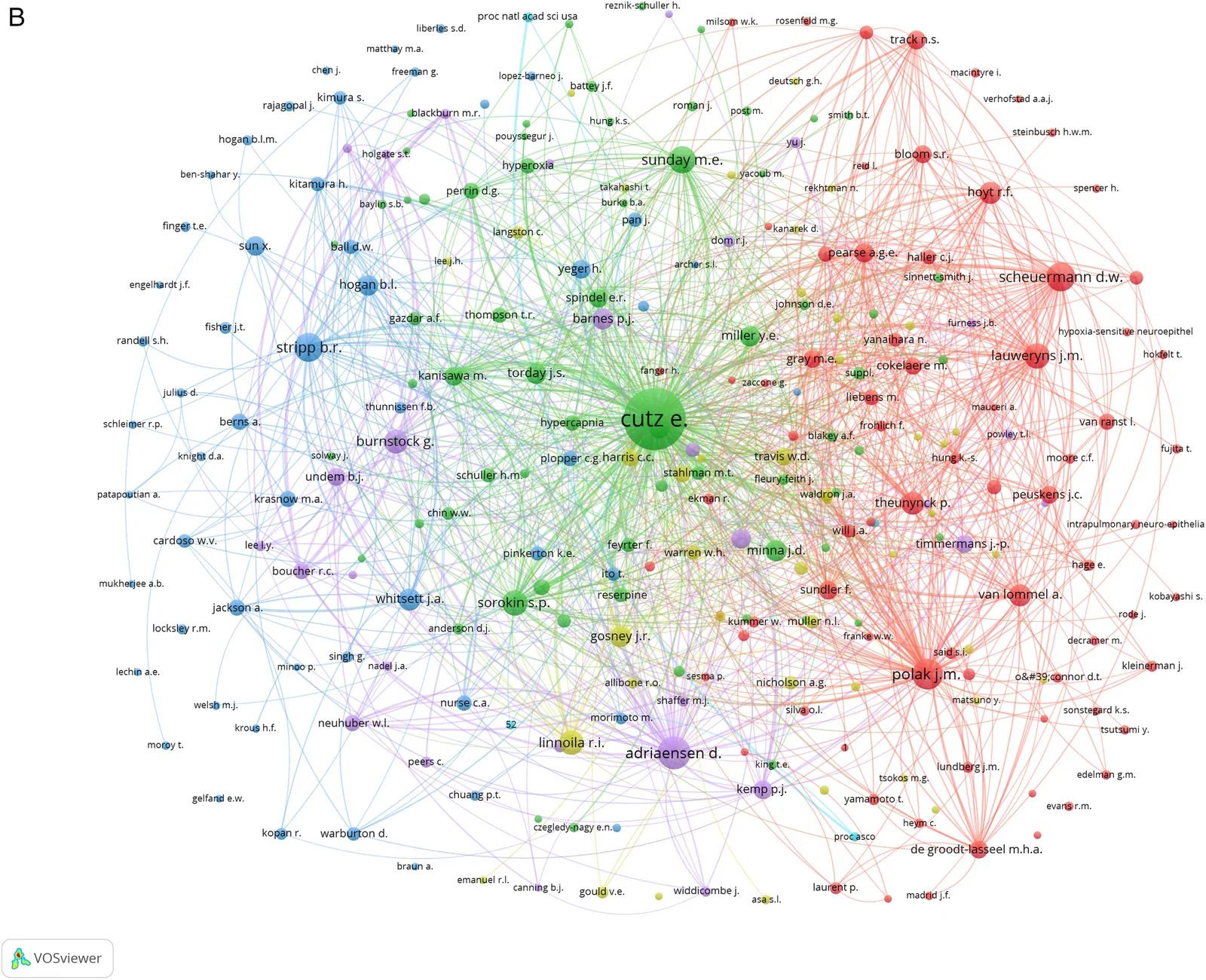


**Figure:**
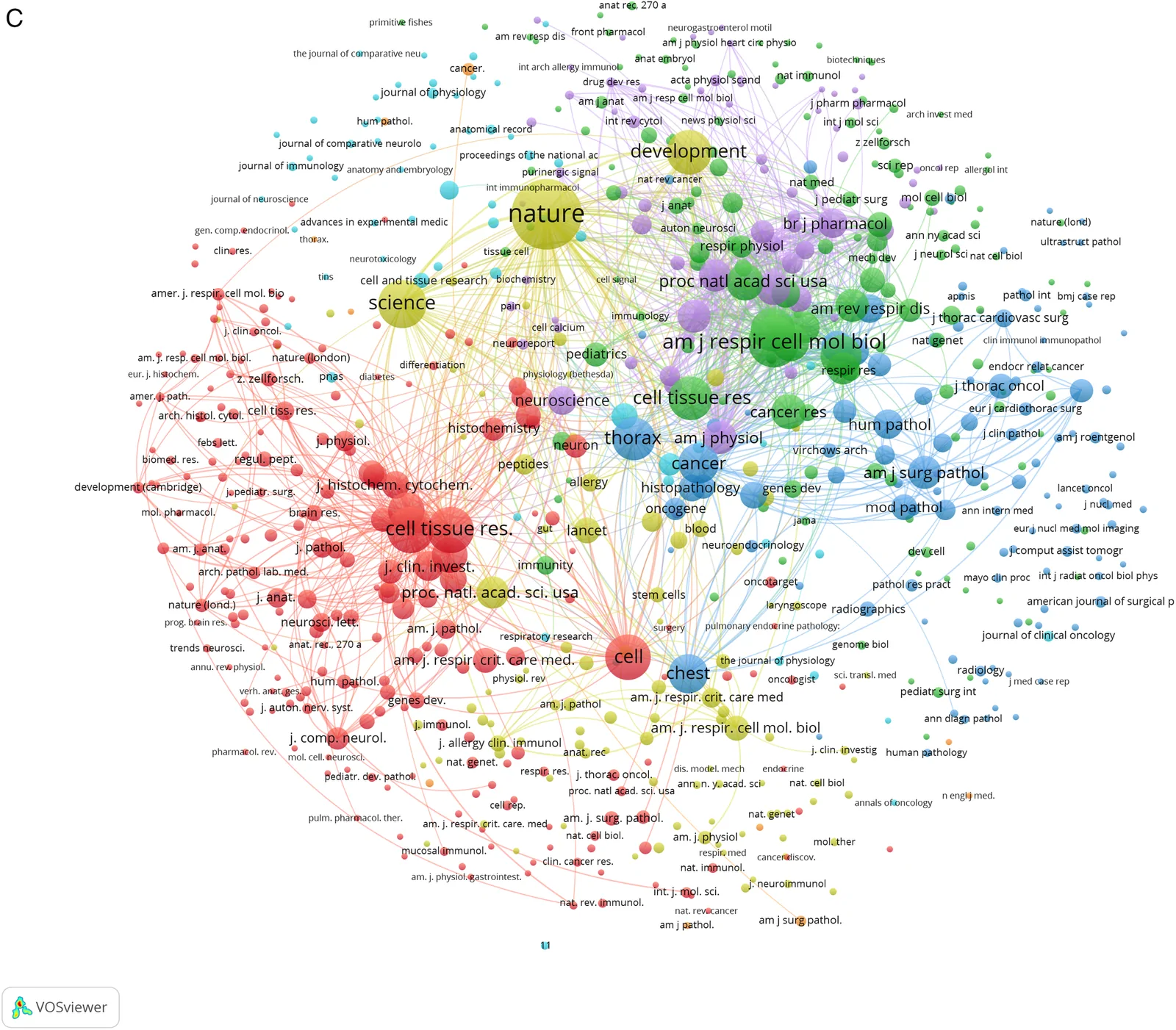


### 3.4 Co-cited authors and journal knowledge sources

The cited-author co-citation network showed a structure distinct from the co-authorship network (Figure 3B). Cutz E formed the largest node, accompanied by prominent co-cited authors including Adriaensen D, Sunday M, Polak J, Lauweryns J, Burnstock G, Linnoila R, Scheuermann D, Stripp B, and Pearse AGE. The network contained several densely connected clusters of authors whose publications were repeatedly cited together.

The included articles were published across respiratory medicine, anatomy, histology, pathology, physiology, oncology, and general biomedical journals (Supplementary Table 1E). American Journal of Respiratory Cell and Molecular Biology published the largest number of articles (*n* = 28), followed by The Anatomical Record (*n* = 22), American Journal of Respiratory and Critical Care Medicine (*n* = 15), Cell and Tissue Research (*n* = 14), and Histochemistry and Cell Biology (*n* = 12). The remaining journals in the top 10 were Microscopy Research and Technique (*n* = 11), American Journal of Physiology-Lung Cellular and Molecular Physiology (*n* = 10), Journal of Histochemistry & Cytochemistry (*n* = 8), Histology and Histopathology (*n* = 8), and American Journal of Pathology (*n* = 7).

In the source co-citation network, American Journal of Respiratory Cell and Molecular Biology ranked first with 1,707 co-citations, followed by American Journal of Respiratory and Critical Care Medicine with 1,272 and American Journal of Pathology with 1,098. European Respiratory Journal and Proceedings of the National Academy of Sciences of the United States of America received 1,030 and 1,023 co-citations (Figure 3C; Supplementary Table 1E). The remaining sources in the top 10 were The Anatomical Record (821 co-citations), Science (666), Mechanisms of Development (655), Cancer (538), and Journal of Clinical Investigation (526).

### 3.5 Keyword hotspots and thematic structure

The most frequently occurring keyword was “lung,” with 94 occurrences, followed by “neuroepithelial bodies” with 42, “pulmonary neuroendocrine cells” with 40, “neuroendocrine cells” with 34, and “immunohistochemistry” with 33 (Figure 4A; Supplementary Table 1F). The remaining terms in the top 10 were “carcinoid” and “neuroendocrine” (22 occurrences each), “asthma” and “DIPNECH” (21 each), and “bombesin” (19). Terms related to inflammation, chemosensation, epithelial biology, pulmonary development, hyperplasia, and neuroendocrine neoplasia were also represented in the keyword network.

**Figure 4:**
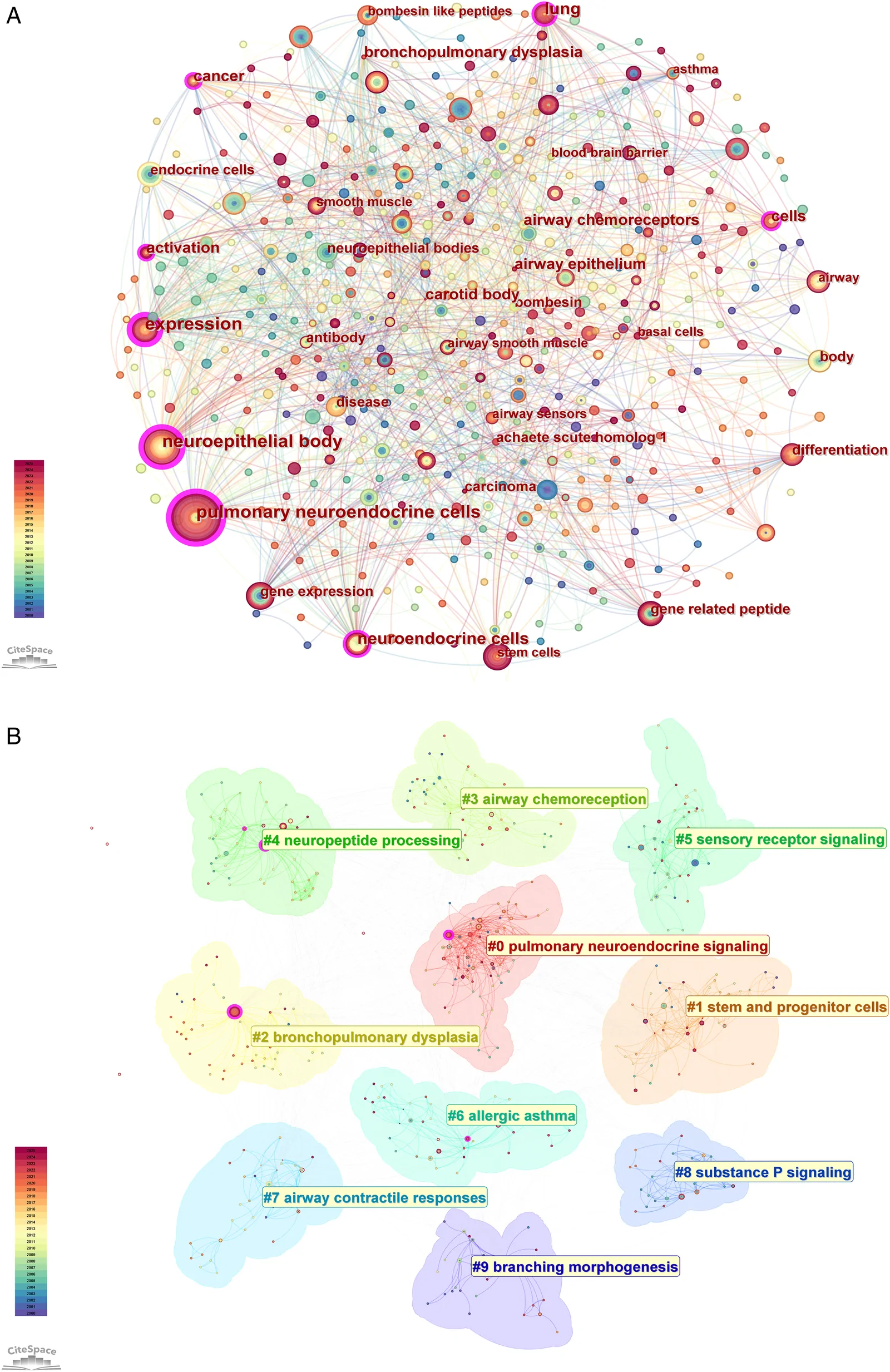
Keyword co-occurrence and thematic clustering in PNEC/NEB research at the airway neuroimmune interface from 1990 to 2025. **(A)** Keyword co-occurrence network generated using CiteSpace. Node size reflects occurrence frequency, node colors represent the temporal distribution of keywords, and links indicate co-occurrence relationships. **(B)** Keyword clustering network generated using CiteSpace. Initial cluster labels were generated using the log-likelihood ratio algorithm and refined according to representative terms without altering cluster membership; clusters were numbered in descending order of size.

The CiteSpace keyword clustering network showed a modularity *Q* of 0.5172, indicating meaningful separation between thematic clusters, and a weighted mean silhouette score of 0.8012, indicating good within-cluster consistency. Ten major thematic clusters were identified (Figure 4B). Cluster **#0 pulmonary neuroendocrine signaling** formed the largest cluster and contained terms associated with PNECs, NEBs, neuroendocrine differentiation, and secretory mediators. Cluster **#1 stem and progenitor cells** represented research concerning epithelial plasticity, progenitor properties, and regenerative responses. Cluster **#2 bronchopulmonary dysplasia** contained terms related to neonatal lung disease, hypoxia, and developmental pulmonary abnormalities, whereas cluster **#3 airway chemoreception** was centered on airway sensing and neuroepithelial-body physiology. Cluster **#4 neuropeptide processing** included terms associated with peptide precursors, convertases, and neuroendocrine secretion, and cluster **#5 sensory receptor signaling** represented receptor-mediated sensory and neural communication.

Disease- and response-related themes were represented by cluster #6 allergic asthma, cluster #7 airway contractile responses, and cluster #8 substance P signaling. Cluster #9 branching morphogenesis constituted a pulmonary-development-related group. Collectively, the cluster structure delineated interconnected domains involving pulmonary neuroendocrine signaling, airway sensing, developmental regulation, epithelial plasticity, neuropeptide pathways, allergic inflammation, and airway functional responses.

### 3.6 Temporal evolution and emerging research fronts

The keyword timeline showed that the identified themes developed over partially overlapping periods rather than as a single sequential progression (Figure 5A). Early publications were primarily associated with pulmonary neuroendocrine terminology, neuroepithelial bodies, bombesin and other secretory mediators, airway chemoreception, and branching morphogenesis. Developmental and cellular topics, including stem and progenitor cells, bronchopulmonary dysplasia, neuropeptide processing, and sensory receptor signaling, extended across the middle portion of the study period. More recent parts of the timeline contained terms related to allergic asthma, airway contractile responses, substance P signaling, PNEC-specific terminology, epithelial differentiation, and hyperplasia.

**Figure 5:**
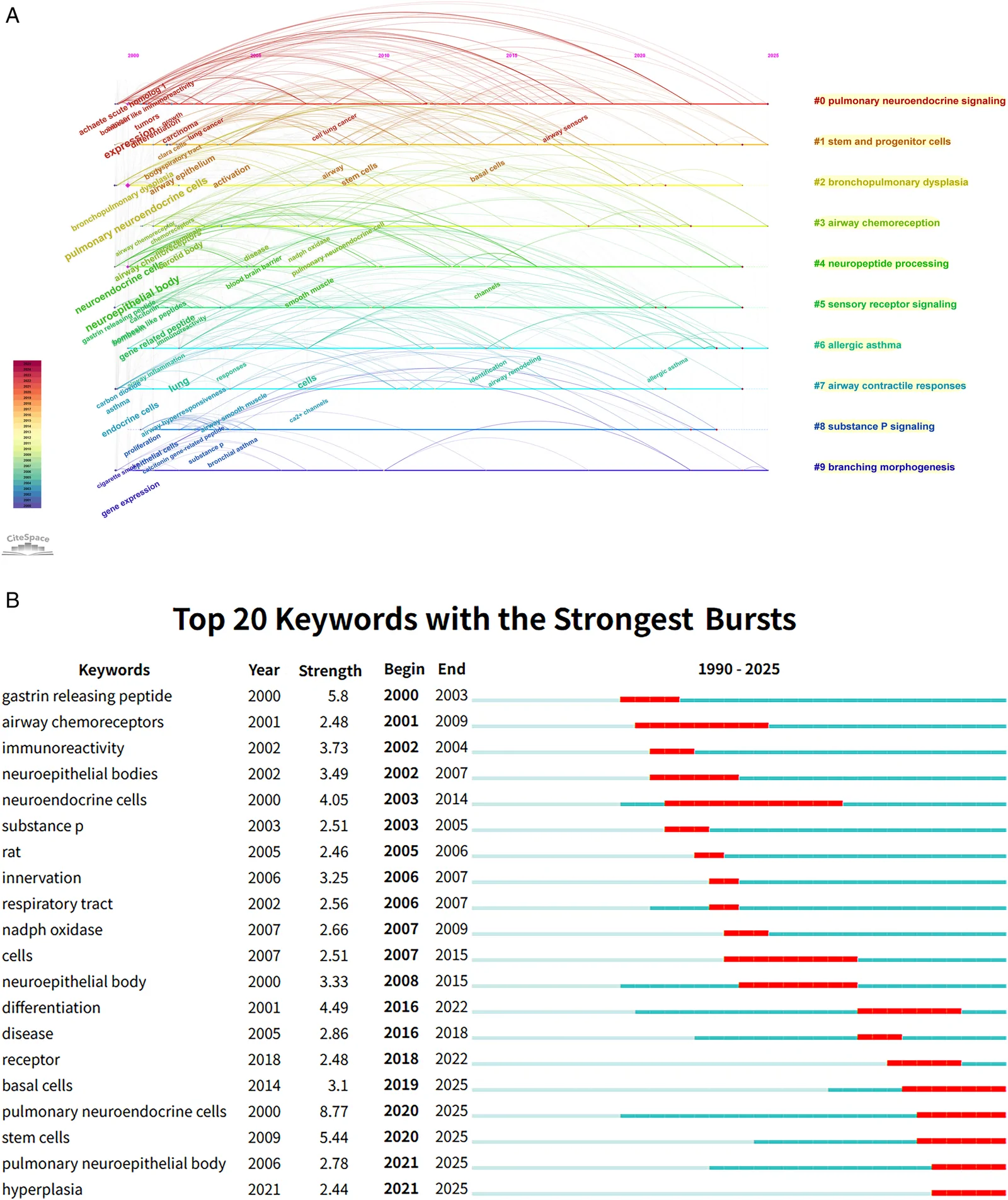
Temporal evolution and burst detection of keywords in PNEC/NEB research at the airway neuroimmune interface from 1990 to 2025. **(A)** Timeline visualization of major keyword clusters. Node position indicates the year of occurrence, and node size reflects frequency. **(B)** Top 20 keywords with the strongest bursts. Red segments indicate burst periods, and strength represents burst intensity.

Keyword burst analysis further identified periods of increased attention to specific topics (Figure 5B). “Gastrin-releasing peptide” showed a burst strength of 5.80 from 2000 to 2003, while “airway chemoreceptors” showed a sustained burst from 2001 to 2009. “Neuroendocrine cells” remained active from 2003 to 2014, and “neuroepithelial body” showed a burst from 2008 to 2015. Later bursts included “differentiation” from 2016 to 2022 and “receptor” from 2018 to 2022. The strongest recent burst was observed for “pulmonary neuroendocrine cells,” with a strength of 8.77 from 2020 to 2025. “Stem cells” also showed a sustained burst from 2020 to 2025, while “pulmonary neuroepithelial body” and “hyperplasia” remained active from 2021 to 2025. These temporal patterns indicated increasing recent attention to PNEC-specific biology, stem-cell-related properties, receptor signaling, differentiation, and hyperplastic changes.

### 3.7 Reference co-citation structure and landmark publications

The reference co-citation network contained several influential publications that were repeatedly cited together (Figure 6A; Table 1). The most frequently co-cited reference was the 2016 study by Branchfield et al., with 19 co-citations and a network degree of 13. The 2013 review by Cutz et al. ranked second with 12 co-citations, followed by the 2012 study by Song et al. with 9 and the 2009 study by Brouns et al. with 8. Studies by De Proost et al., Gorshtein et al., Baral et al., and Nassar et al. each received 5 co-citations, while publications by Schnorbusch et al. and Morrisey et al. each received 4. These references covered airway sensory functions, neuroepithelial-body biology, lung development, purinergic and GABAergic signaling, pulmonary neuroendocrine cell hyperplasia, and neuroimmune-related respiratory responses.

**Figure 6:**
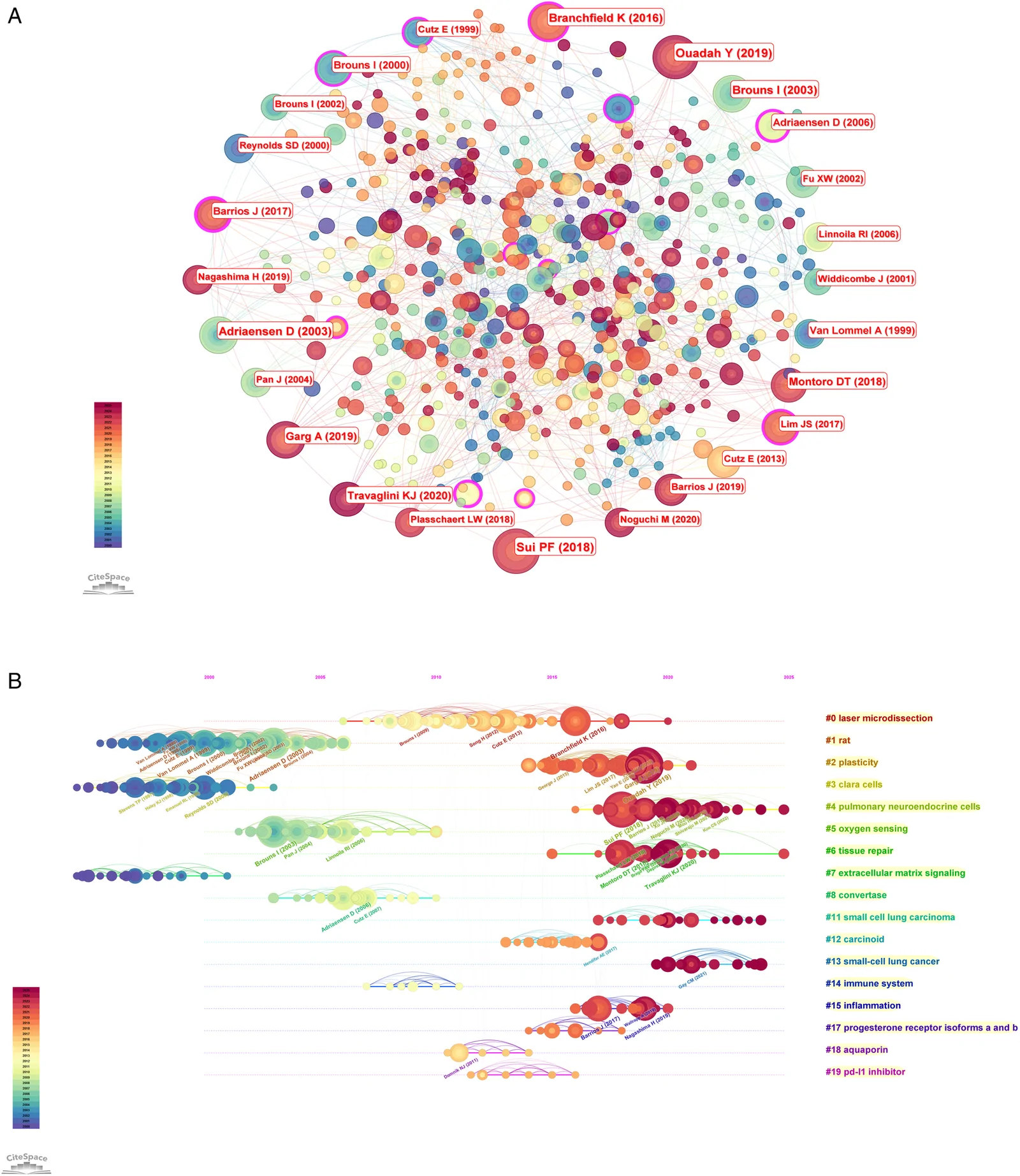
Reference co-citation structure and citation bursts in PNEC/NEB research at the airway neuroimmune interface from 1990 to 2025. **(A)** Reference co-citation network, in which node size reflects co-citation frequency and links represent joint citations. **(B)** Timeline visualization of reference co-citation clusters. **(C)** Top 20 references with the strongest citation bursts; red segments indicate burst intervals.

**Figure:**
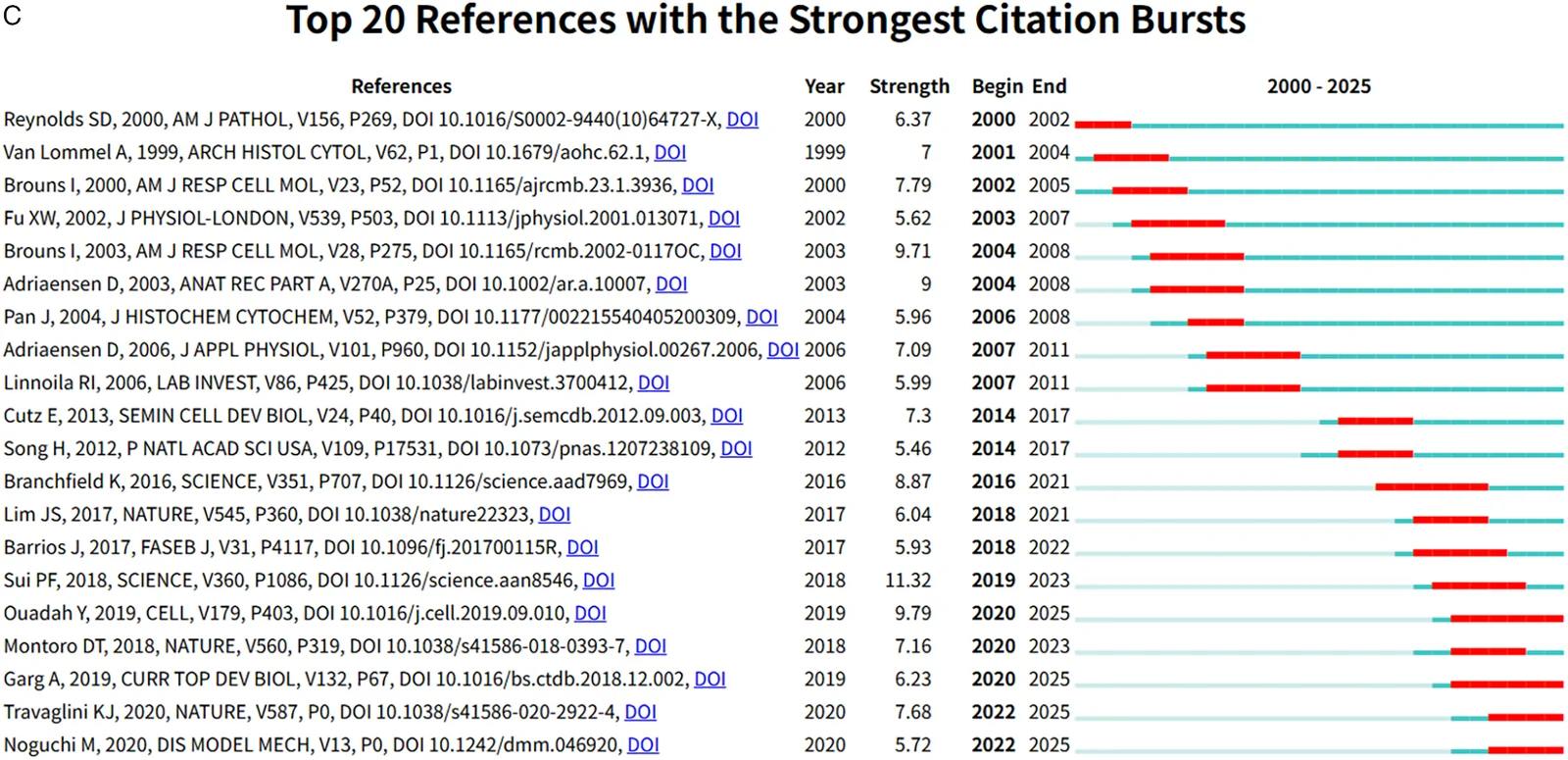


The co-citation timeline identified clusters labeled laser microdissection, rat, plasticity, Clara cells, pulmonary neuroendocrine cells, oxygen sensing, tissue repair, immune system, inflammation, and several neoplastic or treatment-related terms (Figure 6B). Clusters related to structural characterization, innervation, animal models, and oxygen sensing were represented in the earlier part of the timeline. Later clusters included pulmonary neuroendocrine cells, epithelial plasticity, tissue repair, immune system, inflammation, and disease-related terminology.

Reference burst analysis identified successive groups of publications receiving concentrated citation attention (Figure 6C). Earlier burst references included studies by Reynolds, Van Lommel, Brouns, Adriaensen, and Pan. More recent burst references included publications by Branchfield, Sui, Ouadah, and Noguchi, with several continuing into the final years of the study period.

Under the predefined primary citation-count rule, the publication with the highest document-level citation count was *The new World Health Organization classification of lung tumours*, with 895 citations. It was followed by *The molecular basis of lung morphogenesis* with 567 and *Pathology of lung cancer* with 509 (Table 1). Other highly cited publications included *Terminal bronchioles harbor a unique airway stem cell population that localizes to the bronchoalveolar duct junction* with 494 citations, *Regulation of immune responses by the airway epithelial cell landscape* with 460, and *Bronchopulmonary neuroendocrine tumors* with 432. Publications specifically addressing neuroepithelial-body-associated epithelial renewal and regeneration received 427 and 395 citations, respectively. The study on fetal pulmonary neuroendocrine differentiation received 373 citations, while *Pulmonary neuroendocrine cells amplify allergic asthma responses* received 369.

## 4 Discussion

### 4.1 Principal findings and scope

The analysis of the 595-publication corpus showed a gradual expansion of PNEC/NEB research at the intersection of airway sensory, secretory, epithelial, and neuroimmune biology, with annual output increasing notably after 2019 despite year-to-year fluctuations. Keyword co-occurrence, burst detection, and reference co-citation analyses traced a shift within the analyzed literature from neuroendocrine characterization, secretory mediators, NEB organization, and airway chemosensation toward developmental regulation, epithelial plasticity, hyperplasia, and neuroimmune signaling. The newer topics largely developed alongside, rather than replacing, the established sensory and neuroendocrine foundations of the field. The identification of the leading cited publications was robust across database-specific and alternative citation-count approaches, supporting the stability of the principal citation-ranking findings.

Three intersecting lines of research were apparent. One centered on airway chemoreception and neuroendocrine secretion, including bombesin/gastrin-releasing peptide (GRP), serotonin, neuropeptide processing, and receptor signaling. A second focused on lung development and epithelial plasticity, encompassing branching morphogenesis, PNEC differentiation, stem and progenitor populations, tissue repair, and bronchopulmonary dysplasia. The third linked PNECs to disease-associated airway responses through allergic asthma, substance *P*-related signaling, airway contractile responses, inflammation, and hyperplastic change. Collectively, these literature patterns show that PNECs have increasingly been studied as specialized epithelial sensors linking neuroendocrine secretion with developmental, reparative, and immune processes in the airway; this interpretation was further contextualized using representative experimental studies (11).

### 4.2 Historical foundations of PNEC research

Early PNEC research was shaped by studies of NEB morphology, sensory innervation, oxygen responsiveness, and neuroendocrine products such as bombesin and serotonin. The prominence of Cutz, Adriaensen, Lauweryns, and Timmermans in the cited-author network reflects the influence of this work. Morphological investigations defined NEBs as organized clusters of neuroendocrine epithelial cells closely associated with complex nerve endings, while anterograde tracing demonstrated vagal afferent innervation and strengthened the anatomical basis for their proposed sensory role (12, 13).

Seminal physiological studies established NEBs as oxygen-sensitive airway chemoreceptors, with reduced oxygen availability altering membrane excitability and K⁺ conductance (3). Subsequent work implicated NADPH oxidase in this oxygen-sensing process through modulation of O₂-sensitive K⁺ currents (14). Mechanical stretch was also found to stimulate serotonin release from PNECs through mechanosensitive pathways, linking changes in airway mechanics to local neuroendocrine secretion (15). By combining environmental sensing, neural connectivity, and mediator release, this early work defined the sensor–secretory model that later guided investigations into epithelial regulation, tissue repair, and airway inflammation.

### 4.3 Development, progenitor states, and epithelial plasticity

Developmental and regenerative studies formed another sustained branch of the PNEC literature, reflected in the clusters related to stem and progenitor cells, bronchopulmonary dysplasia, and branching morphogenesis. Genetic lineage tracing enabled PNEC behavior to be followed during lung development, epithelial injury, and tumorigenesis (16). Neuroendocrine specification depends on the transcription factor ASCL1, whereas Notch signaling regulates PNEC abundance and coordinates neuroendocrine differentiation with the distribution of secretory and ciliated epithelial lineages (6, 17). During airway morphogenesis, dispersed PNECs migrate toward branch points and assemble into NEBs, accounting for their characteristic localization within the developing bronchial tree (18).

The NEB microenvironment was subsequently recognized as a specialized epithelial niche. After airway injury, NEB-associated regions retain pollutant-resistant progenitor populations capable of participating in localized epithelial regeneration (19). Clara-like precursors have also been identified adjacent to presumptive NEBs during development, indicating a close spatial relationship between neuroendocrine structures and regenerative epithelial populations (20). Lineage-tracing experiments further showed that a rare subset of mature PNECs can adopt reserve stem-cell activity after severe epithelial damage, self-renewing and generating non-neuroendocrine descendants under the control of RB/p53 and Notch pathways (7). This body of work links the bibliometric prominence of differentiation, stem cells, and tissue repair to a context-dependent role for PNECs and the NEB niche in epithelial maintenance and regeneration.

### 4.4 PNECs at the airway neuroimmune interface

The bibliometric analyses placed pulmonary neuroendocrine signaling within a broader network of sensory, inflammatory, and airway-response themes. Neuropeptide processing and sensory receptor signaling formed distinct clusters, whereas allergic asthma, airway contractile responses, and substance P signaling represented more disease- or function-oriented branches. Frequently occurring terms such as bombesin/GRP, serotonin, and CGRP further reflected sustained interest in mediator-dependent communication. The literature therefore increasingly addressed PNECs as active participants in airway signaling rather than solely as morphologically defined neuroendocrine cells.

Temporal patterns distinguished this established sensory–secretory foundation from the more recent neuroimmune direction of the field. GRP and airway chemoreceptors showed earlier keyword bursts, while receptor signaling, allergic asthma, airway contractile responses, and PNEC-specific terminology became more prominent in later years. Reference co-citation and burst analyses likewise emphasized studies of purinergic and GABAergic signaling, neuropeptide-dependent immune regulation, and allergic airway inflammation. These bibliometric signals guided the selection of representative studies in Table 2 and the input–PNEC/NEB–mediator–target–response framework presented in Figure 7; the individual biological connections in that framework were drawn from experimental studies rather than inferred from co-occurrence patterns alone.

**Table 2:** Representative mechanistic studies linking pulmonary neuroendocrine cells and neuroepithelial bodies to airway sensing, epithelial regulation, and immune responses. Studies are organized according to the principal physiological or experimental context and summarize the PNEC/NEB-associated mechanism, major cellular target, functional finding, experimental model, and representative publication. The selected studies are illustrative examples used to contextualize the sensory–secretory and neuroimmune themes identified in the bibliometric analysis and summarized conceptually in Figure 7.

Context/input	PNEC/NEB mechanism	Principal target	Main functional finding	Model/evidence	Representative study
Hypoxia	O₂-sensitive K⁺ conductance and membrane excitability	NEB sensory unit/afferent pathway	Transduces reduced oxygen into an airway chemosensory response	Fetal rabbit NEBs; whole-cell patch clamp	Youngson et al., 1993 (3)
Mechanical stretch	Mechanosensitive serotonin (5-HT) release	Local epithelial and neural compartments	Links physical airway expansion to regulated neuroendocrine secretion	Primary fetal rabbit PNECs and H727 cells	Pan et al., 2006 (15)
Air exposure/impaired NEB clustering	Robo1/2-dependent organization; excess neuropeptide production	Pulmonary macrophages and extracellular matrix	Increases immune infiltration and contributes to alveolar simplification	Robo1/2-deficient mice; *in vivo* genetic model	Branchfield et al., 2016 (9)
Allergen exposure	CGRP activates ILC2s; GABA acts on airway epithelium	ILC2s and goblet cells	Amplifies type 2 inflammation and goblet-cell hyperplasia	PNEC-deficient OVA/HDM mouse models; human asthma tissue	Sui et al., 2018 (8)
Severe epithelial injury	Rb/p53/Notch-regulated reserve stem-cell program	Damaged airway epithelium	Rare PNECs self-renew and generate non-neuroendocrine repair clones	Lineage-traced mouse airway injury model	Ouadah et al., 2019 (7)
Luminal succinate	SUCNR1 activation triggers ATP release	Submucosal-gland myoepithelial cells via P2Y1	Induces gland contraction and promotes expulsion of gland contents	Pig and human submucosal glands; *ex vivo* and cellular assays	Yu et al., 2022 (4)
Airway development	Notch receptor dosage and epithelial niche interactions	PNECs, club/secretory cells, and ciliated cells	Controls PNEC abundance, presumptive NEB size, and epithelial distribution	Developing mouse lung; stepwise Notch deletion	Morimoto et al., 2012 (17)

5-HT, 5-hydroxytryptamine; ATP, adenosine triphosphate; CGRP, calcitonin gene-related peptide; GABA, *γ*-aminobutyric acid; HDM, house dust mite; ILC2, group 2 innate lymphoid cell; NEB, neuroepithelial body; OVA, ovalbumin; P2Y1, purinergic receptor P2Y1; PNEC, pulmonary neuroendocrine cell; Rb, retinoblastoma protein; SUCNR1, succinate receptor 1.

**Figure 7:**
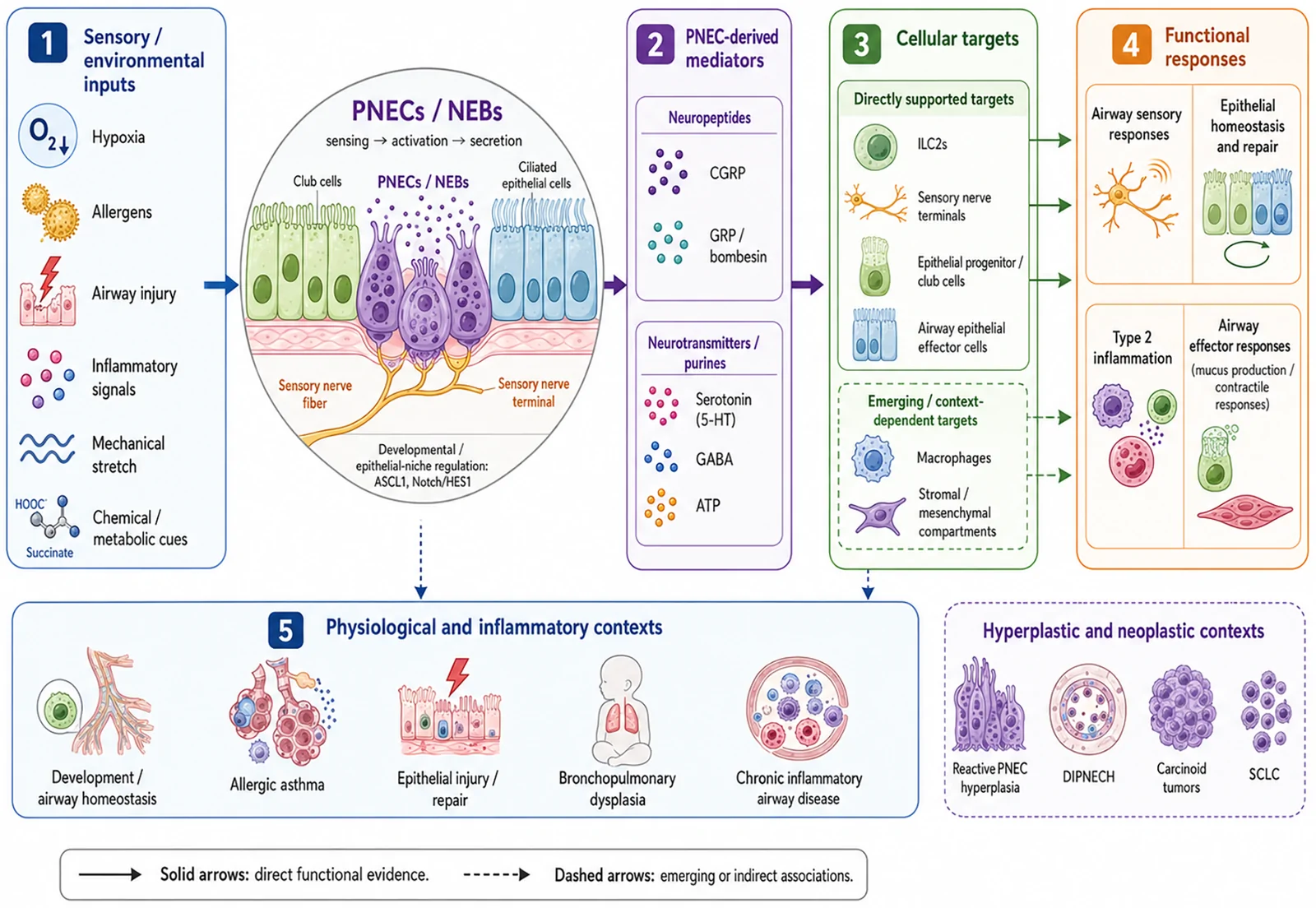
Pulmonary neuroendocrine cells at the airway neuroimmune interface: sensory inputs, mediator outputs, cellular targets, and functional responses. This literature-informed schematic summarizes relationships reported in representative experimental studies used to contextualize the bibliometric findings. These studies describe pulmonary neuroendocrine cells (PNECs) and neuroepithelial bodies (NEBs) as innervated sensory–secretory units associated with environmental sensing, mediator release, and developmental or epithelial-niche regulation. PNEC-derived neuropeptides, neurotransmitters, and purines signal to epithelial, neural, and immune targets, contributing to airway sensory responses, epithelial homeostasis and repair, type 2 inflammation, and airway effector responses. The lower panels summarize the principal physiological, inflammatory, hyperplastic, and neoplastic contexts associated with PNEC research. Solid arrows indicate relationships reported in the cited studies as having direct functional support, whereas dashed arrows indicate emerging or indirectly supported associations. CGRP, calcitonin gene-related peptide; GABA, *γ*-aminobutyric acid; GRP, gastrin-releasing peptide; ILC2, group 2 innate lymphoid cell.

Representative experimental studies have described PNECs and NEBs as sensory–secretory units. Reduced oxygen availability alters NEB activity, and mechanical stretch stimulates serotonin release from PNECs (3, 15). A separate metabolic pathway involves succinate sensing through succinate receptor 1 (SUCNR1), followed by adenosine triphosphate (ATP) release and purinergic receptor P2Y1-dependent contraction of submucosal-gland myoepithelial cells (4). Live-cell imaging has also demonstrated ATP-mediated purinergic communication within the NEB microenvironment (21), while studies in GAD67-GFP reporter mice identified a localized GABAergic signaling system associated with NEBs (22). These representative studies describe stimulus-specific secretory responses through which PNECs may influence nearby neural, epithelial, and contractile compartments.

The most clearly defined immune pathway involves PNEC-derived mediators in allergic and developmental lung responses. In Robo-deficient mice, abnormal NEB organization and increased neuropeptide signaling were accompanied by macrophage accumulation, extracellular-matrix abnormalities, and alveolar simplification (9). In allergic-airway models, PNECs positioned near group 2 innate lymphoid cells (ILC2s) released CGRP that enhanced type 2 immune activity, whereas PNEC-derived GABA promoted goblet-cell hyperplasia (8). Early-life allergen exposure further increased neural stimulation of PNEC secretion and mucus production (23), and primate studies linked pulmonary PNEC-derived GABA to epithelial mucus remodeling (24). More recent work implicated communication between transient receptor potential ankyrin 1 (TRPA1)-expressing vagal neurons and PNECs in allergic inflammation and showed that house-dust-mite exposure induced protease-activated receptor 1 (PAR-1)-dependent CGRP release from human PNEC-enriched cultures (25, 26). These representative studies describe mediator-specific pathways linking PNECs and NEBs with epithelial and type 2 immune responses in particular experimental settings.

### 4.5 Context-dependent airway responses and disease branches

The thematic map extended beyond airway sensing and allergic inflammation to encompass developmental, hyperplastic, and neoplastic disorders. Airway contractile responses and bronchopulmonary dysplasia formed distinct keyword clusters, while hyperplasia remained an active burst term through 2025. Carcinoid tumors, diffuse idiopathic pulmonary neuroendocrine cell hyperplasia (DIPNECH), lung cancer, and small cell lung cancer (SCLC) also appeared in the keyword and co-citation networks. Their parallel representation suggests that PNEC research has developed across several related but biologically distinct disease settings rather than along a single pathological continuum.

The role of PNECs differs substantially among these settings. In neonatal lung disease, bombesin-immunoreactive PNECs were reduced during early hyaline membrane disease but increased in established bronchopulmonary dysplasia, indicating disease-associated changes in PNEC abundance during abnormal postnatal lung development (27). Reactive PNEC proliferations arising in injured lung can be distinguished morphologically and phenotypically from DIPNECH, which is characterized by diffuse neuroendocrine cell proliferation and may occur with airflow obstruction, tumorlets, and pulmonary carcinoids (28, 29). Pulmonary neuroendocrine tumors represent a separate pathological spectrum (10). In genetically engineered mice, combined inactivation of Trp53 and Rb1 showed that pulmonary neuroendocrine cells are a major cell of origin for small cell lung cancer, although other epithelial populations can acquire a neuroendocrine phenotype under specific genetic conditions (30). Normal PNEC physiology, reactive hyperplasia, DIPNECH and carcinoid tumors, and high-grade neuroendocrine carcinoma should therefore be interpreted as distinct branches rather than successive stages of one disease process.

### 4.6 Emerging mechanistic and translational directions

The recent prominence of PNEC-specific terminology, stem cells, differentiation, receptor signaling, and hyperplasia points to cellular heterogeneity as a major unresolved question. PNEC-focused single-cell analysis has identified molecularly distinct neuroendocrine populations (2), while broader human airway atlases have placed these rare cells within the cellular organization of the healthy epithelium (31). Single-cell and spatial transcriptomic profiling of the developing human lung has also resolved the timing and localization of PNEC lineage emergence and inferred potential interactions between epithelial progenitors and adjacent stromal populations (32). Combining these approaches with spatial proteomics, multiplex imaging, and lineage tracing may determine whether solitary PNECs and innervated NEBs differ in their developmental origin, mediator repertoire, or response to injury.

Progress toward translation will also require experimental systems that preserve human epithelial, neural, and immune interactions. Stem-cell-derived lung cultures and airway organoids can generate neuroendocrine populations under controlled conditions and provide platforms for manipulating developmental and disease-associated pathways (1). In human embryonic-stem-cell-derived lung progenitors, inhibition of Notch signaling promoted PNEC differentiation, whereas subsequent suppression of RB1 and TP53 permitted the formation of SCLC-like tumors (33). Future co-culture models should pair PNECs with defined sensory neurons, ILC2s, epithelial progenitors, macrophages, or mesenchymal cells and incorporate mediator- or receptor-specific perturbations. Such experiments could distinguish the actions of CGRP, GABA, serotonin, ATP, and GRP/bombesin and test which circuits represented in Figure 7 and Table 2 remain active in particular human disease settings.

### 4.7 Methodological strengths, analytical scope, and limitations

This study focused on PNECs at the intersection of airway sensing, neuroendocrine secretion, epithelial regulation, and immune responses, an area whose literature is dispersed across respiratory physiology, developmental biology, neurobiology, inflammation, and pathology. The search strategy therefore emphasized neuroimmune, inflammatory, and neuropeptide-related terms rather than all publications mentioning PNECs. This thematic restriction improves alignment between literature retrieval and the study question. However, it may reduce the direct retrieval of foundational studies whose indexed titles, abstracts, or keywords do not contain the required terms, particularly early work on oxygen sensing, developmental regulation, sensory innervation, and epithelial plasticity. Representative foundational studies in these domains were therefore incorporated into the historical and biological interpretation of the bibliometric findings. Integrating WoSCC and Scopus broadened bibliographic coverage, while DOI- and title-based verification supported duplicate identification and bibliographic standardization. Citation indicators were calculated using a predefined Scopus-prioritized, non-summing rule, with WoSCC counts used only for publications not indexed in Scopus. Database-specific and maximum-count sensitivity analyses demonstrated that the identification and ordering of the leading cited publications were robust to this methodological choice. Despite the relatively small size of the field, the 595 publications generated a well-defined thematic structure, supported by a modularity Q of 0.5172 and a weighted mean silhouette score of 0.8012. The combined use of Bibliometrix, VOSviewer, CiteSpace, and OriginPro enabled complementary analyses of productivity, collaboration, co-citation, thematic evolution, and citation bursts, which were further interpreted using representative experimental studies in Figure 7 and Table 2.

Several limitations should be considered. The analysis was limited to English-language articles and reviews indexed in WoSCC or Scopus and therefore does not represent the entire PNEC literature. The modest annual publication volume, database-specific coverage, incomplete author or institutional disambiguation, keyword normalization, and algorithm-dependent clustering may have influenced individual rankings and network patterns. Publications from 2023 to 2025 had substantially less time to accumulate citations than older publications and were therefore inherently disadvantaged in citation-based comparisons; accordingly, their lower total citation counts should not be interpreted as evidence of lower research quality. Citation counts were also database dependent because WoSCC and Scopus differ in source coverage and citation indexing. The direct concordance analysis was therefore restricted to a conservatively matched subset with unambiguous one-to-one record correspondence rather than a complete enumeration of all publications represented in both databases. Nevertheless, the strong cross-database rank correlation, substantial overlap of the database-specific top-10 lists, and unchanged top 10 under the maximum-count approach indicate that the principal citation-ranking conclusions were not materially affected by the selected rule. In addition, bibliometric relationships indicate patterns of scholarly attention rather than biological causality, and the mechanistic framework should be interpreted as an evidence-informed synthesis rather than a systematic review of all experimental pathways.

## 5 Conclusion

Within the interface-focused literature analyzed here, PNEC/NEB research has evolved from morphological characterization, airway chemosensation, and classical neuroendocrine mediators toward developmental regulation, epithelial plasticity, allergic inflammation, and neuroimmune signaling. Research has increasingly focused on PNECs and NEBs as localized sensory–secretory units and on their potential mediator-specific interactions with epithelial, neural, and immune compartments. Future studies using human tissues, organoids, spatial and single-cell approaches, and multicellular co-culture models will be important for resolving PNEC heterogeneity and determining the disease-specific relevance of these proposed signaling relationships.

## Data Availability

The raw data supporting the conclusions of this article will be made available by the authors, without undue reservation.
