# Supplementary material for: Pulmonary neuroendocrine cells at the airway neuroimmune interface: a bibliometric analysis
Source: Front Med (Lausanne). 2026 Oct 14;13:1953372. doi: 10.3389/fmed.2026.1953372 (PMC13713817; doi:10.3389/fmed.2026.1953372)
Supplement: Supplementary file 1 [file Supplementaryfile1.docx]

**Search query**

**WoS Core Collection**

TS=(

("pulmonary neuroendocrine cell*" OR

"airway neuroendocrine cell*" OR

"bronchial neuroendocrine cell*" OR

"lung neuroendocrine cell*" OR

"pulmonary neuroendocrine system" OR

"neuroepithelial bod*" OR

(PNEC* NEAR/3 (lung OR pulmonary OR airway OR bronch*))

)

AND

(neuroimmun* OR "neuro-immune" OR immun* OR inflamm* OR cytokine* OR chemokine* OR "immune regulation" OR "mucosal immunity" OR "innate immunity" OR neuropeptide* OR CGRP OR "calcitonin gene-related peptide" OR

"substance P" OR bombesin OR "gastrin-releasing peptide" OR GRP)

AND

(lung OR pulmonary OR airway OR bronch* OR respiratory)

)

**Scopus**

TITLE-ABS-KEY(

("pulmonary neuroendocrine cell*" OR "airway neuroendocrine cell*" OR

"bronchial neuroendocrine cell*" OR "lung neuroendocrine cell*" OR

"pulmonary neuroendocrine system" OR "neuroepithelial bod*" OR

(PNEC* W/3 (lung OR pulmonary OR airway OR bronch*))

)

AND

(neuroimmun* OR "neuro-immune" OR immun* OR inflamm* OR cytokine* OR chemokine* OR "immune regulation" OR "mucosal immunity" OR "innate immunity" OR

neuropeptide* OR CGRP OR "calcitonin gene-related peptide" OR "substance P" OR bombesin OR "gastrin-releasing peptide" OR GRP )

AND

(lung OR pulmonary OR airway OR bronch* OR respiratory)

)

**PubMed**

("pulmonary neuroendocrine cells"[Title/Abstract] OR

"airway neuroendocrine cells"[Title/Abstract] OR

"neuroepithelial bodies"[Title/Abstract] OR

PNEC[Title/Abstract])

AND

(neuroimmune [Title/Abstract] OR inflammation [Title/Abstract] OR immunity [Title/Abstract] OR CGRP[Title/Abstract] OR "calcitonin gene-related peptide"[Title/Abstract] OR

"substance P"[Title/Abstract] OR neuropeptide*[Title/Abstract])

AND

(COPD[Title/Abstract] OR asthma [Title/Abstract] OR infection [Title/Abstract] OR

Hypoxia [Title/Abstract] OR "airway inflammation"[Title/Abstract] OR lung [Title/Abstract])
