# Supplementary material for: Pulmonary neuroendocrine cells at the airway neuroimmune interface: a bibliometric analysis
Source: Front Med (Lausanne). 2026 Oct 14;13:1953372. doi: 10.3389/fmed.2026.1953372 (PMC13713817; doi:10.3389/fmed.2026.1953372)
Supplement: Supplementary file 2 [file Table1.docx]

**Supplementary Table 1. Detailed bibliometric characteristics of pulmonary neuroendocrine cell research, 1990-2025.**

(The table summarizes the descriptive indicators underlying Results sections 3.1-3.5. All analyses were based on the final dataset of 595 publications.)

**A. Annual publication and citation indicators**

| **1990-2007** | | | | | **2008-2025** | | | | |
| --- | --- | --- | --- | --- | --- | --- | --- | --- | --- |
| **Year** | **Publications** | **Total citations** | **Mean citations/article** | **Mean citations/year** | **Year** | **Publications** | **Total citations** | **Mean citations/article** | **Mean citations/year** |
| 1990 | 7 | 380 | 54.29 | 1.47 | 2008 | 12 | 724 | 60.33 | 3.18 |
| 1991 | 12 | 444 | 37.00 | 1.03 | 2009 | 16 | 966 | 60.38 | 3.35 |
| 1992 | 10 | 298 | 29.80 | 0.85 | 2010 | 15 | 720 | 48.00 | 2.82 |
| 1993 | 24 | 876 | 36.50 | 1.07 | 2011 | 7 | 775 | 110.71 | 6.92 |
| 1994 | 18 | 437 | 24.28 | 0.74 | 2012 | 17 | 855 | 50.29 | 3.35 |
| 1995 | 15 | 380 | 25.33 | 0.79 | 2013 | 16 | 600 | 37.50 | 2.68 |
| 1996 | 13 | 470 | 36.15 | 1.17 | 2014 | 14 | 669 | 47.79 | 3.68 |
| 1997 | 25 | 754 | 30.16 | 1.01 | 2015 | 24 | 918 | 38.25 | 3.19 |
| 1998 | 9 | 332 | 36.89 | 1.27 | 2016 | 14 | 508 | 36.29 | 3.30 |
| 1999 | 9 | 558 | 62.00 | 2.21 | 2017 | 12 | 284 | 23.67 | 2.37 |
| 2000 | 16 | 1,832 | 114.50 | 4.24 | 2018 | 12 | 911 | 75.92 | 8.44 |
| 2001 | 17 | 1,966 | 115.65 | 4.45 | 2019 | 17 | 476 | 28.00 | 3.50 |
| 2002 | 14 | 1,078 | 77.00 | 3.08 | 2020 | 24 | 1,131 | 47.12 | 6.73 |
| 2003 | 12 | 605 | 50.42 | 2.10 | 2021 | 27 | 1,142 | 42.30 | 7.05 |
| 2004 | 20 | 661 | 33.05 | 1.44 | 2022 | 26 | 488 | 18.77 | 3.75 |
| 2005 | 12 | 437 | 36.42 | 1.66 | 2023 | 19 | 147 | 7.74 | 1.93 |
| 2006 | 23 | 1,480 | 64.35 | 3.06 | 2024 | 28 | 330 | 11.79 | 3.93 |
| 2007 | 17 | 646 | 38.00 | 1.90 | 2025 | 22 | 121 | 5.50 | 2.75 |

Notes: Total citations are cumulative citations received by each publication-year cohort up to June 30, 2026. Scopus citation counts were used for publications indexed in Scopus, whereas WoSCC counts were used only for publications not indexed in Scopus; counts were not summed. Mean citations/year was calculated using the citable years available for each cohort.

**B. Country output and international collaboration**

| **Rank** | **Country** | **SCP** | **MCP** | **Total articles** | **MCP ratio** |
| --- | --- | --- | --- | --- | --- |
| 1 | United States | 204 | 14 | 218 | 6.4% |
| 2 | Canada | 52 | 5 | 57 | 8.8% |
| 3 | Belgium | 54 | 0 | 54 | 0.0% |
| 4 | Japan | 40 | 3 | 43 | 7.0% |
| 5 | United Kingdom | 32 | 7 | 39 | 17.9% |
| 6 | China | 27 | 2 | 29 | 6.9% |
| 7 | Italy | 24 | 4 | 28 | 14.3% |
| 8 | Germany | 21 | 3 | 24 | 12.5% |
| 9 | Spain | 13 | 2 | 15 | 13.3% |
| 10 | Poland | 14 | 1 | 15 | 6.7% |
| 11 | Netherlands | 7 | 1 | 8 | 12.5% |
| 12 | France | 8 | 0 | 8 | 0.0% |
| 13 | Australia | 4 | 3 | 7 | 42.9% |
| 14 | Turkey | 5 | 2 | 7 | 28.6% |
| 15 | Norway | 4 | 1 | 5 | 20.0% |
| 16 | Portugal | 5 | 0 | 5 | 0.0% |
| 17 | Sweden | 3 | 1 | 4 | 25.0% |
| 18 | Switzerland | 3 | 1 | 4 | 25.0% |
| 19 | Korea | 1 | 2 | 3 | 66.7% |
| 20 | Egypt | 3 | 0 | 3 | 0.0% |

Abbreviations: MCP, multiple-country publication; SCP, single-country publication. MCP ratio = MCP/total articles.

| **C. Leading institutions** | | |  | **D. Leading authors** | | | | |
| --- | --- | --- | --- | --- | --- | --- | --- | --- |
| **Rank** | **Institution** | **Articles** |  | **Rank** | **Author** | **Articles** | **Total citations** | **Local h-index** |
| 1 | University of Toronto | 40 |  | 1 | Cutz E | 47 | 1,836 | 27 |
| 2 | University of Antwerp | 32 |  | 2 | Adriaensen D | 41 | 1,546 | 22 |
| 3 | Harvard University | 26 |  | 3 | Timmermans J | 40 | 1,425 | 22 |
| 4 | Harvard Medical School | 20 |  | 4 | Brouns I | 32 | 1,308 | 21 |
| 5 | Harvard University Medical Affiliates | 20 |  | 5 | Sunday M | 27 | 1,254 | 19 |
| 6 | University of California | 20 |  | 6 | Yeger H | 26 | 1,026 | 17 |
| 7 | Baylor College of Medicine | 17 |  | 7 | Pan J | 21 | 816 | 16 |
| 8 | Duke University | 14 |  | 8 | Pintelon I | 20 | 621 | 16 |
| 9 | Hospital for Sick Children | 13 |  | 9 | Lauweryns J | 18 | 298 | 9 |
| 10 | Massachusetts General Hospital | 12 |  | 10 | Ito T | 17 | 756 | 12 |
| 11 | National Cancer Institute | 12 |  | 11 | Scheuermann D | 15 | 443 | 10 |
| 12 | Collegium Medicum, Nicolaus Copernicus University | 10 |  | 12 | Kitamura H | 12 | 625 | 8 |
| 13 | University of California San Diego | 10 |  | 13 | Udaka N | 11 | 616 | 8 |
| 14 | Brigham and Women's Hospital | 9 |  | 14 | De P I | 11 | 459 | 11 |
| 15 | Memorial Sloan Kettering Cancer Center | 9 |  | 15 | Van L A | 10 | 287 | 7 |
| 16 | University of California System | 9 |  | 16 | Van G J | 9 | 394 | 9 |
| 17 | University of Colorado System | 9 |  | 17 | Burnstock G | 8 | 885 | 8 |
| 18 | Vanderbilt University Medical Center | 9 |  | 18 | Sun X | 8 | 740 | 5 |
| 19 | Helmholtz Association | 8 |  | 19 | Miller Y | 8 | 433 | 8 |
| 20 | Justus Liebig University Giessen | 8 |  | 20 | Linnoila R | 8 | 341 | 7 |

Notes: Institution names follow the standardized affiliation labels retained in the dataset; broad university systems and affiliated organizations may therefore appear as separate entries. Author citation counts and h-indices were calculated within the final 595-publication dataset; the h-index is a local rather than career-wide indicator.

**E. Productive and co-cited journals**

| **Rank** | **Productive journal** | **Articles** | **Co-cited journal** | **Co-citations** |
| --- | --- | --- | --- | --- |
| 1 | *Am J Respir Cell Mol Biol* | 28 | *Am J Respir Cell Mol Biol* | 1,707 |
| 2 | *Anat Rec* | 22 | *Am J Respir Crit Care Med* | 1,272 |
| 3 | *Am J Respir Crit Care Med* | 15 | *Am J Pathol* | 1,098 |
| 4 | *Cell Tissue Res* | 14 | *Eur Respir J* | 1,030 |
| 5 | *Histochem Cell Biol* | 12 | *Proc Natl Acad Sci U S A* | 1,023 |
| 6 | *Microsc Res Tech* | 11 | *Anat Rec* | 821 |
| 7 | *Am J Physiol Lung Cell Mol Physiol* | 10 | *Science* | 666 |
| 8 | *J Histochem Cytochem* | 8 | *Mech Dev* | 655 |
| 9 | *Histol Histopathol* | 8 | *Cancer* | 538 |
| 10 | *Am J Pathol* | 7 | *J Clin Invest* | 526 |
| 11 | *Proc Natl Acad Sci U S A* | 7 | *Clin Chest Med* | 514 |
| 12 | *Lab Invest* | 7 | *Nat Rev Immunol* | 460 |
| 13 | *FASEB J* | 7 | *Am J Physiol Lung Cell Mol Physiol* | 436 |
| 14 | *Pediatr Pulmonol* | 7 | *Genes Dev* | 397 |
| 15 | *Respir Physiol Neurobiol* | 6 | *Development* | 373 |
| 16 | *Cancer Res* | 5 | *J Histochem Cytochem* | 369 |
| 17 | *Lung* | 5 | *Respir Physiol Neurobiol* | 351 |
| 18 | *Eur Respir J* | 4 | *Microsc Res Tech* | 349 |
| 19 | *Science* | 4 | *Cell Tissue Res* | 342 |
| 20 | *J Clin Invest* | 4 | *Lab Invest* | 341 |

Notes: Journal productivity was measured by the number of included articles. Source co-citation counts reflect how often journals were cited together within the dataset and do not represent the citation count of articles published by the journal.

**F. Most frequent author keywords**

| **Rank** | **Keyword** | **Frequency** | **Rank** | **Keyword** | **Frequency** |
| --- | --- | --- | --- | --- | --- |
| 1 | Lung | 94 | 16 | Pulmonary Neuroendocrine Cell | 11 |
| 2 | Neuroepithelial Bodies | 42 | 17 | Diffuse Idiopathic Pulmonary Neuroendocrine Cell Hyperplasia | 10 |
| 3 | Pulmonary Neuroendocrine Cells | 40 | 18 | Innervation | 10 |
| 4 | Neuroendocrine Cells | 34 | 19 | Mouse | 10 |
| 5 | Immunohistochemistry | 33 | 20 | Inflammation | 9 |
| 6 | Carcinoid | 22 | 21 | Lung Cancer | 9 |
| 7 | Neuroendocrine | 22 | 22 | Atypical Carcinoid | 8 |
| 8 | Asthma | 21 | 23 | Bronchopulmonary Dysplasia | 8 |
| 9 | DIPNECH | 21 | 24 | Chemoreceptors | 8 |
| 10 | Bombesin | 19 | 25 | Development | 8 |
| 11 | Serotonin | 19 | 26 | Endocrine Cells | 8 |
| 12 | Rat | 17 | 27 | Epithelium | 8 |
| 13 | Lung Development | 15 | 28 | Small Cell Lung Cancer | 8 |
| 14 | Calcitonin gene-related peptide | 13 | 29 | Stem Cells | 8 |
| 15 | Immunocytochemistry | 12 | 30 | Airway | 7 |

Notes: Frequencies were calculated from author keywords after the thesaurus-based standardization used in the bibliometric analysis. Terms are shown as retained in the final analytical dataset.

Abbreviations: DIPNECH, diffuse idiopathic pulmonary neuroendocrine cell hyperplasia; PNEC, pulmonary neuroendocrine cell; WoSCC, Web of Science Core Collection.
